# Geranium Oil Nanoemulsion Delivers More Potent and Persistent Fumigant Control of *Callosobruchus maculatus* in Stored Grain

**DOI:** 10.3390/foods14203514

**Published:** 2025-10-15

**Authors:** Samar Sayed Ibrahim, Ameya D. Gondhalekar, Kurt Ristroph, Dieudonne Baributsa

**Affiliations:** 1 Department of Entomology, Purdue University, West Lafayette, IN 47907, USA; si.sayed@nrc.sci.eg (S.S.I.); ameygon@ufl.edu (A.D.G.); 2 Pests and Plant Protection Department, National Research Centre, Giza 12622, Egypt; 3 Department of Agricultural & Biological Engineering, Purdue University, West Lafayette, IN 47907, USA; ristroph@purdue.edu

**Keywords:** post-harvest management, insect pests, grain storage, biopesticides, nanotechnology

## Abstract

Plant essential oils offer eco-friendly alternatives to insecticides, though their instability limits effectiveness. This study evaluated the physicochemical stability and fumigant efficacy of geranium (*Pelargonium graveolens*) oil nanoemulsion (GONE) versus bulk geranium oil (GOB) against *Callosobruchus maculatus*. Geranium oil nanoemulsions (GONEs) were prepared via spontaneous emulsification using 8% oil and varying surfactant levels. The 10% surfactant formulation produced the most uniform and stable nanoemulsion, with an average droplet size of 91.85 ± 0.02 nm and a low polydispersity index of 0.16 ± 0.02. No significant changes in droplet size were observed after 30 days of storage at room temperature and 9 °C, confirming the formulation’s stability. A fumigant bioassay was conducted using five concentrations (50, 100, 150, 200, and 250 µL/L air) of GOB and GONE over 24, 48, 72, and 96 h. Both forms exhibited concentration- and time-dependent toxicity against *C. maculatus*. Complete mortality was achieved sooner and at lower doses with GONE (72 h at 150 µL/L air versus 250 µL/L air for GOB; 96 h at 150 µL/L air for GONE versus 200 µL/L air for GOB). Geranium oil nanoemulsion consistently produced lower LC_50_ and LC_90_ values, indicating greater potency. It also significantly reduced progeny development. Residual fumigant bioassays at the LC_90_ level showed that GONE retained efficacy against *C. maculatus* adults longer than GOB, causing 50% mortality 12 days post-treatment compared to 21% for GOB. Overall, nanoformulation enhanced the potency and persistence of geranium oil, highlighting its promise for protecting stored grains from *C. maculatus*.

## 1. Introduction

With the world’s population expected to reach over 9 billion by 2050, there is an urgent need to increase food production and reduce post-harvest losses to guarantee food security, particularly in developing countries [1]. In these countries, legume crops such as cowpea (*Vigna unguiculata*) are valuable because they provide an inexpensive source of protein, vital nutrients, and a source of income for smallholder farmers [2]. However, post-harvest losses in legume crops such as cowpea, chickpea, peas, green gram, lentils, and common beans are a significant challenge, particularly due to damage caused by various pest beetle species [3].

The cowpea bruchid, *Callosobruchus maculatus* (F.), is a cosmopolitan beetle widely recognized for its damaging effects on stored legumes [4]. Stored legumes undergo quantitative and qualitative damage caused by the feeding of *C. maculatus* larvae [5]. *Callosobruchus maculatus* beetles can reduce stored grain quantity by 5–10% in temperate climates and 20–30% in tropical regions. Within three to four months, up to 60% of the grains in storage will be lost if no control measures are implemented [6,7].

Chemical insecticides and phosphine/aluminum phosphide fumigants are the most common methods for controlling pest beetles. Even though these chemicals may work well, their prolonged utilization and misuse pose significant risks to human health and the environment, increase the likelihood of pest resistance development, and lead to the accumulation of chemical residues in food products [3,8,9]. Moreover, resistance of *C. maculatus* to commonly used synthetic insecticides threatens the effectiveness of conventional pest control methods [10]. Hence, there is a need to explore safer alternatives to chemical control.

Because of their broad-spectrum bioactivity, low environmental persistence, and relative safety for non-target organisms, essential oils (EOs) extracted from aromatic plants have shown great promise as environmentally friendly substitutes for synthetic insecticides [11]. Previous studies have demonstrated the insecticidal actions of different essential oils on stored product insects [12,13,14,15]. In particular, geranium (*Pelargonium graveolens*) essential oil has demonstrated strong insecticidal and repellent effects against a variety of storage insect pests, for instance, *Tribolium castaneum*, *Rhyzopertha dominica*, and *Sitophilus oryzae* [16,17,18].

Studies have identified geraniol and citronellol as the primary constituents of geranium essential oil [19]. Similarly, an analysis of oil extracted from geranium plants cultivated in Egypt found citronellol, geraniol, δ-selinene, and citronellyl formate to be the dominant components [16]. These compounds exhibit strong insecticidal activity against a range of stored-product pests, underscoring their potential as natural alternatives to synthetic fumigants [20].

Beyond pest control, geranium essential oil has extensive applications in the pharmaceutical, cosmetic, and food industries, as well as in traditional remedies and aromatherapy [21]. It is recognized as safe by the European Food Safety Authority (EFSA) for use as a feed additive and holds Generally Recognized As Safe (GRAS) status from the U.S. FDA, indicating minimal risk to consumers and the environment under normal use conditions. Economically viable and widely cultivated, geranium oil also shows promise as a source of safer anti-inflammatory agents [22], reinforcing its value as a sustainable botanical option for pest management.

Essential oils act as fumigants by penetrating the insect’s respiratory and nervous systems, disrupting neural signaling and cellular balance, and ultimately causing paralysis and death [11]. However, their high volatility, poor water solubility, and environmental instability limit broader application [23]. Nanotechnology offers a solution through nanoemulsions—kinetically stable mixtures of water, oil, and surfactant (10–1000 nm)—which enhance stability, bioavailability, and dispersion, thereby improving efficacy while reducing application rates [24,25,26].

Using specialized mechanical equipment, high-energy techniques such as microfluidization, high-pressure homogenization, and ultrasonication can produce nanoemulsions [27]. Lower-energy methods to induce spontaneous emulsification, such as phase inversion methods, phase inversion composition method, and emulsion inversion point method, are inexpensive and can be implemented using simple equipment to produce the NEs [28]. This approach is preferred due to benefits such as increased stability, simplicity and scalability, energy efficiency, and suitability for encapsulating essential oils sensitive to higher temperatures [29]. However, low-energy systems may have certain disadvantages, such as the need for comparatively high surfactant-to-oil ratios during the production of NEs [30].

Previous studies have extensively explored spontaneous emulsification as a low-energy method for producing nanoemulsions [31,32]. These formulations, particularly those incorporating essential oils with insecticidal activity, have demonstrated their effectiveness against various insect species [33,34]. However, formulation parameters, especially the surfactant-to-oil ratio, significantly influence the physicochemical properties and stability of nanoemulsions, which in turn affect their biological activity.

This study focused on formulating geranium oil nanoemulsions (GONE) using a low-energy spontaneous emulsification method. The objective was to identify the minimum effective surfactant concentration required to produce a stable nanoemulsion with reduced particle size. Three surfactant-to-oil ratios were tested, incorporating a co-surfactant (propylene glycol) and a carrier oil (soybean oil), to evaluate their effect on the physical characteristics and stability of the resulting nanoemulsions. In addition to synthesizing and characterizing the formulations, this study aimed to: (i) evaluate the fumigant toxicity of GONE against *C. maculatus* adults in comparison with bulk geranium oil (GOB), (ii) assess their effects on progeny development, and (iii) determine the persistence of both formulations.

While the use of spontaneous emulsification for essential oil-based insecticidal nanoemulsions is well-documented, few studies have systematically evaluated how formulation parameters, especially the surfactant-to-oil ratio, affect their properties and efficacy. Research is also limited on the fumigant activity and residual effects of GONE against *C. maculatus*. This study addresses these gaps by optimizing formulation conditions and comparing the performance of nanoemulsified and bulk geranium oil in pest control under storage conditions, demonstrating that nanoemulsion achieves higher insecticidal potency with reduced oil content.

## 2. Materials and Methods

This research was conducted from October 2024 to March 2025 at Purdue University (West Lafayette, IN, USA).

### 2.1. Experimental Preparation

The organic geranium essential oil used in this study was purchased from Aura Cacia (Norway, IA, USA). This geranium essential oil was extracted via steam distillation of the leaves and flowering branches of *Pelargonium graveolens* (F: Geraniaceae) cultivated in Egypt. Soybean oil (SBO) was purchased from Thermo Fisher Scientific Chemicals, Inc. (Ward Hill, MA, USA), Tween-80 was purchased from MP Biomedicals (Solon, OH, USA), and propylene glycol was purchased from Ward’s Science (West Henrietta, NY, USA).

#### 2.1.1. Nanoemulsion Preparation

Geranium oil nanoemulsion was produced using spontaneous emulsification [29] with some modifications. In this technique, two phases were formed: an organic phase and an aqueous phase. The organic phase was produced by mixing 8 g of geranium oil, 4 g of SBO (carrier oil), and different amounts of the surfactant Tween-80 (5, 10, or 15 g) using a magnetic stirrer (300 rpm) for 5 min. Mixtures of 3 g of propylene glycol (co-surfactant) and different amounts of distilled water (80, 75, or 70 g) were prepared to form the aqueous phase. These concentrations were selected based on preliminary experiments. The organic phase was titrated at 2 mL/min into the aqueous phase under magnetic stirring (600 rpm) for 30 min at an ambient temperature (25 °C) to produce three nanoemulsion formulations, F1, F2, and F3, with different concentrations of Tween-80: 5, 10, and 15%, respectively. The prepared nanoemulsions were examined for their particle size to determine an optimal formulation for additional bioassays. The optimal nanoemulsion formulation was stored for 30 days at 9 °C and 25 °C for further stability testing. A control formulation corresponding to the optimal nanoemulsion was prepared by omitting geranium oil, while maintaining the relative proportions of the other components (the surfactant, co-surfactant, carrier oil, and water). This was done to assess any potential toxic effects attributable to the formulation additives.

#### 2.1.2. Particle Size Characterization

Particle size characterization of the produced nanoemulsions was performed using dynamic light scattering (Nano ZS, Malvern, Worcestershire, UK) at 25 °C. To determine the average size distribution and polydispersity index, each sample was diluted in 1:10 (*v*/*v*) with Milli-Q (MQ) ultrapure water to avoid multiple scattering effects and adjust the suspension viscosity to that of water [35]. A 633 nm laser was used to measure the samples at a scattering angle of 173°, with an average of 11 runs lasting 10 s each. Three replicates were used for the measurements. The same measurement was performed after 30 days for the stored samples to assess product stability.

#### 2.1.3. Insect Rearing

Insect culture of cowpea bruchid (*Callosobruchus maculatus*) was maintained without exposure to insecticides in a Conviron insect growth chamber (Model CMP4030, CONVIRON, Winnipeg, MB, Canada) under controlled conditions of 25 ± 1 °C and 50 ± 5% humidity. Insects were reared on sterilized cowpea seeds in glass jars (2 L) covered by a muslin cloth for ventilation. Uninfested black-eyed cowpea (*Vigna unguiculata*) seeds were purchased from MBS SEED, Inc. (Denton, TX, USA). To disinfect the seeds from any field carried infestation, the product was kept at −20 °C in a deep freezer for more than a week before being used.

A group of *C. maculatus* adults (>500 individuals) obtained from the stock culture using a vacuum aspirator was introduced into separate glass jars (2 L) containing 500 g of cowpea seeds. Adults were allowed to oviposit for 3 days, after which they were removed. The infested seeds were maintained until adult emergence from the subculture. Adults of similar ages (1–3 days old) were used for the experiments. The shape, size, and pattern of the abdomen and elytra were used to distinguish female and male cowpea bruchids. Females are oval in shape, with elytra longer than 1 mm and a larger, black-marked plate at the end of the abdomen. Males, on the other hand, are more rounded, with elytra shorter than 1 mm and a smaller, strip-less plate.

### 2.2. Experimental Setup

#### 2.2.1. Fumigant Toxicity

Insect mortality was assessed by fumigating *C. maculatus* adults in 60 mL glass vials using filter papers treated with bulk geranium oil (GOB) or geranium nanoemulsion (GONE). The samples of GOB and GONE were loaded on the Whatman No.1 filter paper (4.25 cm) at five concentrations: 3, 6, 9, 12, and 15 µL/60 mL air, which is equivalent to 50, 100, 150, 200, and 250 µL/L air, respectively. The control group received no treatment. Assessments were conducted at four exposure intervals—24, 48, 72, and 96 h—each using a separate set of replicate vials. In addition to the control, the five selected concentrations were replicated five times for each time interval (30 replicate vials for each time interval and a total of 120 replicates). Sample-loaded filter papers were fixed on the inner side of the screw caps of the vials without any direct contact with the seeds. Fifteen cowpea seeds and five pairs of sexed *C. maculatus* adults (five females and five males) were placed in each vial. Then, all the vials were closed with the sample-loaded screw caps. Parafilm was wrapped around the caps to maintain an airtight condition. Fluon was appropriately applied inside each glass vial, close to the top, to prevent insects from contacting the treated filter paper. A nanoemulsion formulation containing only the additives without geranium oil was tested at the highest concentration and maximum exposure time (96 h) to check for any toxic effects.

To assess adult mortality, each group of treatments and its control was examined after 24, 48, 72, and 96 h of treatment. At each interval, the contents of each vial were emptied onto a white sheet of paper to assess the mortality of adult cowpea bruchids. Insects displaying any form of movement were recognized as alive. Those that appeared inactive were gently stimulated using a camel hairbrush and monitored for an additional hour to confirm their mortality.

To assess the effects of GOB and GONE on oviposition and reproductive performance of adult insects exposed to the different concentrations (50, 100, 150, 200, and 250 µL/L air) for varying exposure times (24, 48, 72, and 96 h), the number of eggs deposited on seeds in each vial was counted under a microscope. The average number of eggs laid per female was calculated by dividing the total egg count per vial by five, corresponding to the number of females in each vial. After egg counting, the seeds were transferred into clean, ventilated plastic containers and incubated in a Conviron insect growth chamber (Model CMP4030, CONVIRON, Winnipeg, MB, Canada) under controlled conditions of 25 ± 1 °C and 50 ± 5% humidity to monitor and record adult emergence. The number of *C. maculatus* emerging adults was counted from the twentieth day following the start of treatments until no adult emergence was observed (approximately 25 days after the emergence of the first adults). The adult insects that emerged were removed after each evaluation.

#### 2.2.2. Persistence Bioassay

The persistence of the GOB and GONE formulations was evaluated using a concentration corresponding to the previously estimated LC_90_ value, obtained after two days of fumigation. The LC_90_ level was chosen because it represents the concentration at which either the bulk oil or nanoemulsion achieves near-complete insect mortality. The appropriate amount of each formulation was applied to the filter paper as previously described. The treated filter papers were affixed to the inner surface of the vial screw caps. After the insects were introduced, each vial was sealed with a screw cap containing the sample, and the cap was wrapped with Parafilm to ensure an airtight seal. A thin layer of Fluon was applied to the inner upper surface of each vial to prevent the insects from coming into contact with the treated filter paper.

Beginning on the day of treatment, ten unsexed *C. maculatus* adults were introduced into each 60 mL vial every two days. The control groups received no oil treatment. All treatments and control were replicated five times. Persistence was evaluated based on insect mortality, which was recorded 48 h after each group of insect introduction. The process was repeated until the formulations no longer exhibited insecticidal activity. Insects showing movement were recorded as alive, while inactive ones were gently prodded with a camel hairbrush and observed for one hour to confirm death.

### 2.3. Data Analysis

The average droplet size distribution and polydispersity index (PDI) of the nanoemulsion formulations were analyzed using one-way ANOVA followed by Tukey’s HSD test (*p* < 0.05). Mortality and adult emergence data were arcsine square-root transformed, and the mean number of eggs laid per female was square-root transformed to improve normality. Independent samples t-tests were used to compare the bulk essential oil with its nanoemulsion at each concentration and exposure time. At the same time, one-way ANOVA with Tukey’s HSD test (*p* < 0.05) was applied to compare all treatments and controls across exposure times. Lethal concentrations (LC_50_ and LC_90_) were estimated by probit analysis [36]. Probit regression assumptions were evaluated. A significant slope parameter confirmed the linearity of the log-dose response, and variance homogeneity was verified using the Pearson goodness-of-fit test. Differences between LC values were assessed by comparing 95% confidence intervals. All analyses were performed using SPSS software (version 20.0; IBM Corp., Armonk, NY, USA).

## 3. Results

### 3.1. Characterization of Geranium Oil Nanoemulsion

The average size distribution and polydispersity index (PDI) of prepared formulations (F1, F2, and F3) varied depending on the surfactant concentrations (Table 1, Figure S1a). There were significant differences in the particle size (F = 372.59, *p* < 0.001) and PDI (F = 20.67, *p* = 0.002) of the prepared formulations. The smallest particle size was observed in the F2 formulation, composed of 10% surfactant (Tween-80). The formulation F1 with the lowest surfactant concentration (5%) produced the largest particle size (Table 1). Despite significant differences, all three formulations had very low PDI values. Visual inspection revealed clear phase separation in F1 (5% Tween-80) (Figure S2). Based on the particle size results, F2 was the optimal formulation to examine for stability and fumigant toxicity against *C. maculatus* adults. After 30 days of storage, the particle size of F2 formulations was not significantly altered (F = 2.08, *p* = 0.20) when stored at 9 °C or 25 °C (Table 1, Figure S1b). On the other hand, PDI values of F2 after 30 days of storage were significantly reduced when stored at 9 °C (F = 4.79, *p* = 0.05).

### 3.2. Fumigant Toxicity

#### 3.2.1. Insect Mortality

The mortality rates of *C. maculatus* adults exposed to bulk geranium essential oil (GOB) and its nanoemulsion (GONE) significantly increased with concentration and exposure time (Table 2 and Table S1). Geranium oil nanoemulsion (GONE) was generally more effective than GOB, particularly at prolonged exposures. At the lowest concentration (50 µL/L air), mortality increased from 18–20% at 24 h to 78% (GOB) and 89% (GONE) at 96 h, with significant differences between formulations appearing only at 96 h. Geranium oil nanoemulsion achieved significantly higher mortality at 100 and 150 µL/L air than GOB at 96 h and from 72 h onward, respectively. At 200 µL/L air, both formulations caused high mortality, but GONE outperformed at 48 and 72 h. Compared to GOB (96 h at ≥200 µL/L air), GONE (72 h at 150 µL/L air) reached complete mortality earlier. While both treatments resulted in considerably greater mortality (*p* < 0.001), the mortality rate in controls remained low (Table S2). Mortality in the additive-only group did not differ significantly from the negative control (no treatment), confirming that the surfactant and other additives did not contribute to observed insecticidal effects (Table S2). A significant effect of exposure time on mortality within each treatment was observed (Table S1), confirming that time was a critical factor in insecticidal performance. At all concentrations, across time, mortality plateaued by 72 h, with no further significant increase at 96 h, except for GONE at 100 µL/L air (Table 2).

#### 3.2.2. The Concentration-Mortality Response

The GONE treatment consistently exhibited lower LC_50_ and LC_90_ values compared to GOB at all exposure intervals, indicating higher toxicity (Table 3). Notably, a significantly lower LC_90_ value was observed for GONE at 96 h, underscoring its superior efficacy at higher mortality thresholds. The progressive decline in LC values from 24 to 96 h for both formulations further reflects the time-dependent nature of their toxic effects.

#### 3.2.3. Progeny Development

**Figure 1:**
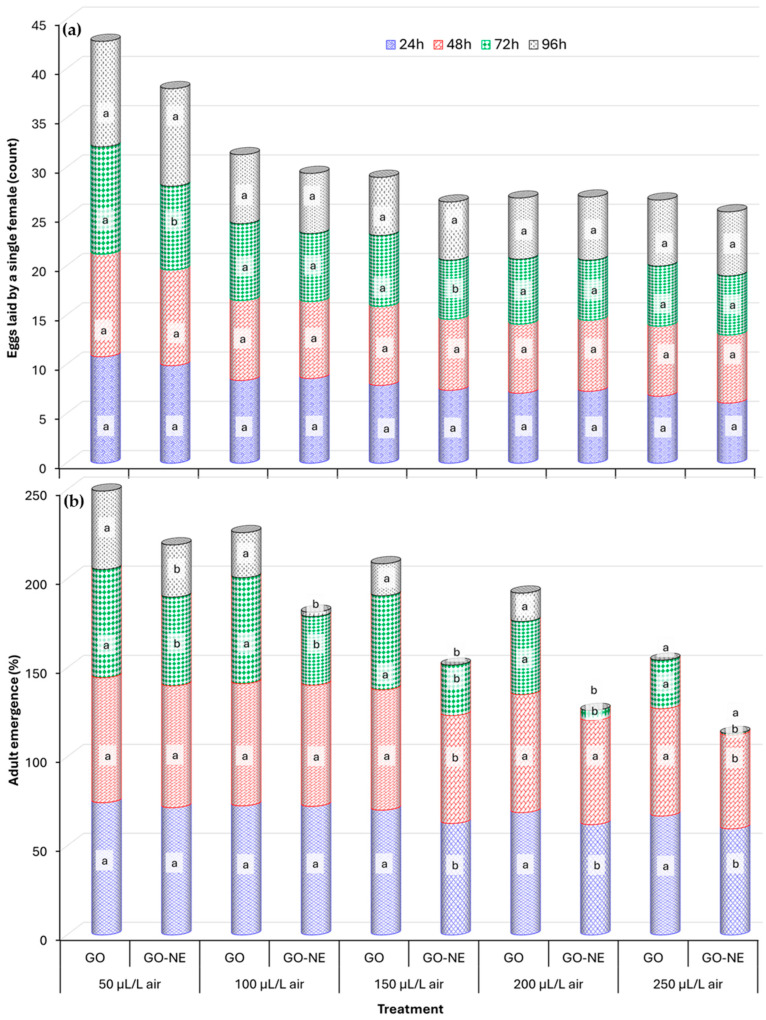
Reproductive effects of Geranium Oil (GOB) and its Nanoemulsion (GONE) on *C. maculatus*: Average (**a**) egg production by a single female and (**b**) adult emergence up to 45 days post-treatment across concentrations and exposure times. Under the same concentration group and exposure time, means followed by the same letters are not significantly different (*p* ˂ 0.05).

The reproduction of *C. maculatus*, evaluated by the number of eggs laid per female and the percentage of adult emergence, was significantly affected by GOB and GONE across all tested concentrations and exposure times (Figure 1 and Table S3). Egg-laying decreased with increasing concentrations of both treatments at each time point. Egg laying increased steadily with exposure time in the control group, peaking at 96 h (Table S4). Both GOB and GONE caused a strong, concentration and time-dependent reduction, with oviposition significantly lower than controls at all concentrations (Table S4). Females exposed to GONE laid considerably fewer eggs compared to those treated with GOB only at 72 h for 50 and 150 µL/L air (Figure 1). At higher concentrations (≥100 µL/L air) and longer exposure times (48–96 h), egg-laying plateaued, and no significant differences were detected among concentrations within the same treatment under the same exposure time (Table S3).

Throughout all exposure times, the control group continuously showed high adult emergence rates. On the other hand, emergence was inhibited by both treatments in a concentration and time-dependent manner (Table S4). Although no significant differences were detected in oviposition rates between GOB and GONE, the percentage of adult emergence varied significantly between the two treatments across most tested concentrations and exposure times (Figure 1 and Table S3). Geranium oil nanoemulsion was more effective than GOB at 72 h at all concentrations and 96 h for all concentrations except at 250 µL/L air. Complete inhibition was achieved at 96 h for both treatments at 250 µL/L air and for GONE at 200 µL/L air.

#### 3.2.4. Persistence

**Figure 2:**
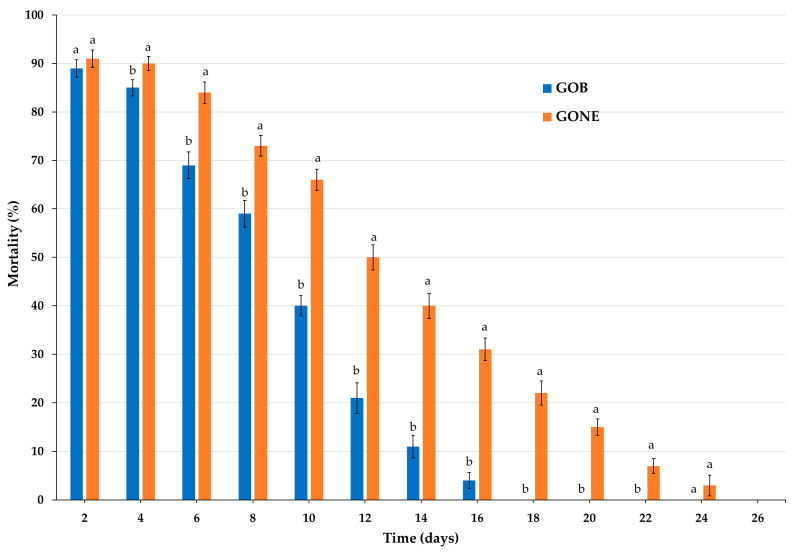
Persistent fumigant toxicity of geranium essential oil (GOB) and geranium oil nanoemulsion (GONE) at LC_90_ on *C. maculatus* adults. Under the same time (days), means followed by the same letters are not significantly different (*p* < 0.05). Significant differences were tested by comparing means using a *t*-test (*p* < 0.05).

The residual fumigant toxicity of geranium essential oil (GOB) and its nanoemulsion formulation (GONE) at LC_90_ was evaluated against *C. maculatus* adults over 24 days (Figure 2, Table S5). On Day 2, both formulations exhibited high mortality rates, with no significant difference (*t* = −0.767, *p* = 0.453). However, from Day 4 onward, significant differences emerged, with GONE consistently demonstrating higher efficacy. By Day 4, GONE maintained 90.0% mortality compared to 85.0% for GOB (*t* = −2.135, *p* = 0.04). The performance gap widened over time: on Day 6, GONE caused 84.0% mortality while GOB dropped to 69.0% (*t* = −4.291, *p* < 0.001). This trend continued through Day 10, where mortality for GONE remained substantially higher compared to GOB (*t* = −8.231, *p* < 0.001). From Day 12 to Day 20, the difference became more pronounced, with GONE maintaining moderate activity while GOB’s efficacy declined sharply, reaching 0.0% mortality by Day 18. Notably, on Day 18, GONE still achieved a 22.0% mortality rate, a significant improvement over GOB (*t* = −12.933, *p* < 0.001).

## 4. Discussion

This study demonstrated that using 10% Tween 80 yielded the most favorable results among the tested nanoemulsions. The concentration of surfactant played a crucial role in reducing the interfacial tension between the oil and aqueous phases, which in turn facilitated the formation of smaller and more stable nanoemulsion droplets [37,38]. The addition of soybean oil as a carrier and water-soluble co-surfactant propylene glycol in the procedure promoted the development of stable nanoparticles and the propensity for small particles to form through the spontaneous emulsification process [31,39].

The quality and uniformity of nanoemulsions are typically characterized by mean droplet diameter and the polydispersity index (PDI), with lower PDI values (<0.25) indicating homogeneous distribution, better dispersion, and good colloidal stability [40,41]. The nanoemulsions obtained in this study had droplet sizes lower than 100 nm, relatively lower than the particle size of comparable surfactants: EO ratios, and very low PDI (0.16), indicating the stability and homogeneity of the formulation (uniformly dispersed). The stability test revealed that changes in the particle size of the optimal nanoemulsion were minor when stored at 25 °C or 9 °C. These findings align with earlier reports where essential oil nanoemulsions stabilized with Tween 80 showed small droplet sizes and low PDI, with higher Tween 80 concentrations further reducing droplet size while maintaining acceptable PDI ranges [32,42]. The results of our study indicate an efficient formulation process that supports the potential of nanoemulsions for improved biological activity and stability.

The bioassay results demonstrate a significant insecticidal potential of GOB and its nanoemulsion (GONE) formulations against *C. maculatus* adults. Mortality increased with concentration and exposure time across all treatments. Geranium oil nanoemulsion (GONE) consistently exhibited higher mortality than GOB, particularly at prolonged exposures, despite containing a substantially reduced oil mass—more than ten times that of equivalent volumes of bulk oil. Probit analysis results further showed lower LC_50_ and LC_90_ values for GONE than GOB across all time intervals, particularly at 96 h. The lower LC_50_ and LC_90_ values of GONE compared to GOB demonstrate that reduced concentrations can still provide adequate control. This reduction in required oil not only enhances cost-effectiveness but also minimizes residues, volatility losses, and environmental impact, highlighting the practical advantages of nanoemulsion formulations for stored product protection [43].

Previous studies have shown that *C. maculatus* mortality was directly proportional to the concentration of the essential oil nanoemulsion and the duration of exposure [42]. Furthermore, consistent with our findings, research has demonstrated that essential oil nanoemulsions exhibited markedly greater toxicity and fumigant efficacy than their bulk or crude forms [44,45]. Nanoformulation improved the efficacy of geranium oil, likely due to increased surface area of the nano-sized droplets, enhanced dispersion, and bioavailability, resulting in more effective delivery of toxic constituents, thereby accelerating mortality [46].

Our findings indicate that egg laying decreased significantly with increasing concentrations and exposure times for both treatments (GOB, GONE) compared to the control. However, no significant differences were observed between the two treatments across all concentrations and time intervals. This study suggests that the enhanced toxicity of GONE is attributed to improved dispersion and bioavailability, which enables greater penetration through the insect cuticle and spiracles [47,48]. Apart from these physicochemical benefits, nanoemulsions have the potential to decrease volatilization rates, which would extend the duration of toxic vapors in the fumigation chamber [49]. Its increased fumigant activity may also suppress mating or egg-laying through knockdown effects, consistent with reports of oviposition inhibition by pure and nanoformulated essential oils against stored product insect pests [44,45,50].

Though fumigated females were able to lay eggs, a significant proportion of these eggs failed to emerge as adults, suggesting that nanoemulsions enhance the ability of essential oils to disrupt insect development. Thus, the enhanced toxicity of the GONE may be attributed not only to improved dispersion and bioavailability but also to deeper or faster penetration and prolonged vapor persistence. Consistent with our findings, previous studies reported population declines, reduced egg hatchability, and inhibited progeny production in stored-product pests following treatment with essential oil nanoemulsions, attributed to their disruption of insect biochemical, metabolic, behavioral, and physiological processes [44,51,52].

The bioassay data on the residual fumigant toxicity of both GOB and GONE at LC_90_ against *C. maculatus* adults over 26 days demonstrated that GONE had prolonged efficacy compared to the non-formulated form (GOB). This extended persistence of GONE can be attributed to its enhanced physicochemical properties, such as reduced volatility and improved stability, which allow the active compounds to remain effective over extended periods [47]. As a result, insects experience more prolonged exposure to toxic vapors compared to bulk essential oils [38,41]. Previous studies have reported similar patterns of prolonged toxicity and stability in essential oils and their nanoformulations. For example, *A. conyzoides*, *A. fragrantissima*, and *T. minuta* essential oils showed extended efficacy against *C. maculatus* [45]. Likewise, nanoformulated *Cuminum cyminum* and *Mentha longifolia* essential oils retained fumigant activity and exhibited slow release with greater stability than their pure forms [53,54]. These findings support the superior potential of nanoformulations in controlling *C. maculatus* during grain storage.

## 5. Conclusions

This study demonstrates that geranium essential oil is an effective fumigant against *C. maculatus*, with the nanoemulsion formulation providing superior and longer-lasting insecticidal activity than the bulk oil while requiring substantially less active material. Incorporating just 8% oil into the nanoemulsion reduced the total oil mass by more than tenfold compared to the bulk formulation, without compromising efficacy—potentially lowering both the environmental impact and cost. Among the tested formulations, the F2 (10% Tween-80) showed the highest performance, achieving greater mortality, stronger progeny suppression, and prolonged residual activity. The use of plant-derived geranium oil enhances safety by minimizing chemical residue concerns, supporting its potential as a sustainable botanical alternative for stored-legume protection. Further research should evaluate its performance under real storage conditions, optimize large-scale production, assess cost-effectiveness, and investigate ecological safety and effects on non-target organisms.

## Figures and Tables

**Table 1:** Particle size (d.nm) and polydispersity index (PDI) of different formulations (F1, F2, and F3) geranium oil nanoemulsion (GONE) formulations and the F2 formulation stored at 9 and 25 °C for 30 days.

Formulation Characterization				Formulation 2 (F2) Storage		
Formulation	Particle Size (d.nm)	PDI		Storage Time	Particle Size (d.nm)	PDI
F1 ^†^	126.03 ± 0.98 a *	0.16 ± 0.004 a		Initial	91.85 ± 1.02 a	0.16 ± 0.02 a
F2	91.85 ± 0.02 c	0.16 ± 0.02 a		9 °C for 30 days	92.17 ± 0.87 a	0.06 ± 0.03 b
F3	113.66 ± 0.63 b	0.06 ± 0.002 b		25 °C for 30 days	94.35 ± 0.91 a	0.10 ± 0.008 ab

^†^ F1, F2, and F3 are GONE formulations that contain 5, 10, and 15% Tween-80, respectively. * All data are means ± standard error of means (SEM). Within the same column, means followed by the same letters are not significantly different (*p* ˂ 0.05).

**Table 2:** Average mortality (%) of *C. maculatus* adults exposed to bulk geranium essential oil (GOB) and geranium oil nanoemulsion (GONE) across various and exposure times.

	Average Mortality (%)			
Treatment	24 h	48 h	72 h	96 h
GOB-50 µL/L air	18.00 ± 2.00 aC *	41.00 ± 1.79 aB	73.00 ± 4.22 aA	78.00 ± 3.59 bA
GONE-50 µL/L air	20.00 ± 1.49 aC	42.00 ± 2.49 aB	83.00 ± 2.13 aA	89.00 ± 1.79 aA
GOB-100 µL/L air	46.00 ± 2.21 aC	65.00 ± 2.23 aB	80.00 ± 2.98 aA	84.00 ± 2.21 bA
GONE-100 µL/L air	45.00 ± 1.67 aD	69.00 ± 2.76 aC	88.00 ± 2.90 aB	97.00 ± 1.52 aA
GOB-150 µL/L air	56.00 ± 2.21 aB	70.00 ± 2.58 aB	86.00 ± 4.52 bA	91.00 ± 2.33 bA
GONE-150 µL/L air	57.00 ± 2.60 aC	75.00 ± 1.66 aB	98.00 ± 1.33 aA	100.00 ± 0.00 aA
GOB-200 µL/L air	64.00 ± 2.21 aB	74.00 ± 3.05 bB	93.00 ± 1.52 bA	96.00 ± 2.21 aA
GONE-200 µL/L air	63.00 ± 2.13 aC	82.00 ± 1.33 aB	100.00 ± 0.00 aA	100.00 ± 0.00 aA
GOB-250 µL/L air	68.00 ± 2.91 aB	76.00 ± 7.91 aB	97.00 ± 1.52 aA	99.00 ± 1.00 aA
GONE-250 µL/L air	70.00 ± 1.49 aC	88.00 ± 1.33 aB	100.00 ± 0.00 aA	100.00 ± 0.00 aA

* All data are means ± standard error of means (SEM). Means followed by the same letters are not significantly different (*p* ˂ 0.05). Lower case letters indicate comparisons between GOB and GONE within each concentration and exposure time. Upper case letters are indicative of comparisons across different exposure times within the same treatment.

**Table 3:** Probit analysis of *C. maculatus* adults’ mortality following exposure to bulk geranium oil (GOB) and geranium oil nanoemulsion (GONE) at different concentrations and exposure times.

Oil Type	Exposure Time (Hours)	LC ^a^	LC Value (LCL ^b^–UCL ^c^)	Slope (SE)	X^2^ (df) ^d^
GOB	24	50	130.45 (113.14–149.81)	1.94 ± 0.24	1.95 (3)
		90	596.35 (431.88–1005.68)		
	48	50	65.88 (46.29–81.98)	1.51 ± 0.23	1.37 (3)
		90	462.28 (326.05–868.01)		
	72	50	22.77 (8.76–36.11)	1.51 ± 0.28	3.93 (3)
		90	159.77 (127.84–225.36)		
	96	50	20.26 (7.69–32.40)	1.69 ± 0.31	4.37 (3)
		90	115.13 (92.91–148.13)		
GONE	24	50	127.65 (110.27–146.85)	1.90 ± 0.24	0.85 (3)
		90	600.39 (432.29–1025.52		
	48	50	61.44 (46.04–74.49)	1.87 ± 0.24	0.92 (3)
		90	296.35 (235.03–426.66)		
	72	50	22.64 (0.00–47.57)	2.39 ± 0.42	8.61 (3)
		90	77.84 (4.55–264.67)		
	96	50	19.86 (6.40–30.35)	2.97 ± 0.72	1.43 (3)
		90	53.56 (38.55–65.76)		

^a^ LC: lethal concentration (µL/L air); ^b^ LCL: Lower confidence level; ^c^ UCL: Upper confidence level; ^d^ Chi-square value (degrees of freedom).

## Data Availability

The original contributions presented in this study are included in the article/Supplementary Materials. Further inquiries can be directed to the corresponding author.

## Supplementary Materials

The following supporting information can be downloaded at: https://www.mdpi.com/article/10.3390/foods14203514/s1, Table S1. Average mortality (%) of *C. maculatus* adults exposed to bulk geranium essential oil (GOB) and geranium oil nanoemulsion (GONE) across various concentrations and exposure times. Table S2. Average mortality (%) of *C. maculatus* adults exposed to bulk geranium essential oil (GOB) and geranium oil nanoemulsion (GONE) across various concentrations and exposure times. Table S3. Reproductive Effects of Geranium Oil (GOB) and Its Nanoemulsion (GONE) on *C. maculatus*: Average Egg Production by a single female and Adult Emergence 45 days post-treatment across concentrations and exposure times. Table S4. Reproductive Effects of Geranium Oil (GOB) and Its Nanoemulsion (GONE) on *C. maculatus*: Average Egg Production by a single female and Adult Emergence 45 days post-treatment across concentrations and exposure times. Table S5. Persistent fumigant toxicity of geranium essential oil (GOB) and geranium oil nanoemulsion (GONE) at LC90 on *C. maculatus* adults. Figure S1. (a) Size distribution by intensity of geranium oil nanoemulsion (GONE) across three formulations; (b) Size distribution by intensity of formulation 2 (F2) during 30 days of storage at different temperatures. Figure S2. Visual appearance of geranium oil nanoemulsion (GONE) formulations (F1, F2, and F3). F1, F2, and F3 correspond to formulations containing 5%, 10%, and 15% Tween-80, respectively. Phase separation was observed in F1.

## References

[1] FAO (2017). The Future of Food and Agriculture: Trends and Challenges.

[2] Metwally E., Sharshar M., Masoud A., Masry A., Fiad A., Kilian B., Sharma S., Shaw P.D., Raubach S., Rakha M. (2021). Development of High Yielding Cowpea [*Vigna unguiculata* (L.) Walp.] Lines with Improved Quality Seeds through Mutation and Pedigree Selection Methods. Horticulturae.

[3] Baributsa D., Lowenberg-Deboer J., Murdock L., Moussa B. Profitable Chemical-Free Cowpea Storage Technology for Smallholder Farmers in Africa: Opportunities and Challenges. Proceedings of the 10th International Working Conference on Stored Product Protection.

[4] Barbosa D.R.e.S., de Oliveira J.V., da Silva P.H.S., Santana M.F., Breda M.O., de França S.M., de Miranda V.L. (2021). Lethal and Sublethal Effects of Chemical Constituents from Essential Oils on *Callosobruchus maculatus* (F.) (Coleoptera: Chrysomelidae: Bruchinae) in Cowpea Stored Grains. J. Plant Dis. Prot..

[5] Gupta H., Deeksha, Urvashi, Reddy S.G.E. (2023). Insecticidal and Detoxification Enzyme Inhibition Activities of Essential Oils for the Control of Pulse Beetle, *Callosobruchus maculatus* (F.) and *Callosobruchus chinensis* (L.) (Coleoptera: Bruchidae). Molecules.

[6] Baoua I.B., Amadou L., Margam V., Murdock L.L. (2012). Comparative Evaluation of Six Storage Methods for Postharvest Preservation of Cowpea Grain. J. Stored Prod. Res..

[7] Seni A., Mishra K.M. (2022). Pulse Beetle, *Callosobruchus* Spp. (Coleoptera: Chrysomelidae); A Major Threat in Legume Grain Storage and Their Management. Acta Phytopathol. Entomol. Hung..

[8] Anaduaka E.G., Uchendu N.O., Asomadu R.O., Ezugwu A.L., Okeke E.S., Chidike Ezeorba T.P. (2023). Widespread Use of Toxic Agrochemicals and Pesticides for Agricultural Products Storage in Africa and Developing Countries: Possible Panacea for Ecotoxicology and Health Implications. Heliyon.

[9] Boyer S., Zhang H., Lempérière G. (2012). A Review of Control Methods and Resistance Mechanisms in Stored-Product Insects. Bull. Entomol. Res..

[10] Gbaye O.A., Oyeniyi E.A., Ojo O.B. (2016). Resistance of *Callosobruchus maculatus* (Fabricius) (Coleoptera: Bruchidae) Populations in Nigeria to Dichlorvos. Jordan J. Biol. Sci..

[11] Isman M.B. (2006). Botanical Insecticides, Deterrents, and Repellents in Modern Agriculture and an Increasingly Regulated World. Annu. Rev. Entomol..

[12] Akbar R., Khan I.A., Alajmi R.A., Ali A., Faheem B., Usman A., Ahmed A.M., El-Shazly M., Farid A., Giesy J.P. (2022). Evaluation of Insecticidal Potentials of Five Plant Extracts against the Stored Grain Pest, *Callosobruchus maculatus* (Coleoptera: Bruchidae). Insects.

[13] Bandi S.M., Mishra P., Venkatesha K.T., Aidbhavi R., Singh B. (2023). Insecticidal, Residual and Sub-Lethal Effects of Some Plant Essential Oils on *Callosobruchus analis* (F.) Infesting Stored Legumes. Int. J. Trop. Insect Sci..

[14] Elbehery H.H., Ibrahim S.S. (2024). Potential Fumigant Toxicity of Essential Oils against *Sitotroga cerealella* (Olivier) (Lepidoptera: Gelechiidae) and Its Egg Parasitoid *Trichogramma evanescens* (Hymenoptera: Trichogrammatidae). Sci. Rep..

[15] Đukić N., Marković T., Mikić S., Čutović N. (2023). Repellent Activity of Basil, Clary Sage and Celery Essential Oils on *Tribolium castaneum* (Herbst). J. Stored Prod. Res..

[16] Abouelatta A.M., Keratum A.Y., Ahmed S.I., El-Zun H.M. (2020). Repellent, Contact and Fumigant Activities of Geranium (*Pelargonium graveolens* L.’Hér) Essential Oils against *Tribolium castaneum* (Herbst) and *Rhyzopertha dominica* (F.). Int. J. Trop. Insect Sci..

[17] Fan G.W., Wang P., Liu Y.S., Sang Y.L., Liu N., Hao Y.J. (2025). Insecticidal Activity of Two *Pelargonium* Essential Oils and Head Transcriptome Analysis of Stored-Product Pest *Tribolium castaneum* (Herbst) (Coleoptera: Tenebrionidae) in Response to Citronellyl Formate Fumigation. Pestic. Biochem. Physiol..

[18] M’hamdi Z., Davì F., Elhourri M., Amechrouq A., Mondello F., Cacciola F., Laganà Vinci R., Mondello L., Miceli N., Taviano M.F. (2024). Phytochemical Investigations, Antioxidant and Insecticidal Properties of Essential Oil and Extracts from the Aerial Parts of *Pelargonium graveolens* from Morocco. Molecules.

[19] Gaire S., Lewis C.D., Booth W., Scharf M.E., Zheng W., Ginzel M.D., Gondhalekar A.D. (2020). Bed Bugs, *Cimex lectularius* L., Exhibiting Metabolic and Target Site Deltamethrin Resistance Are Susceptible to Plant Essential Oils. Pestic. Biochem. Physiol..

[20] Abdelgaleil S.A.M., Gad H.A., Ramadan G.R.M., El-Bakry A.M., El-Sabrout A.M. (2024). Monoterpenes: Chemistry, Insecticidal Activity against Stored Product Insects and Modes of Action—A Review. Int. J. Pest Manag..

[21] Jaradat N., Hawash M., Qadi M., Abualhasan M., Odetallah A., Qasim G., Awayssa R., Akkawi A., Abdullah I., Al-Maharik N. (2022). Chemical Markers and Pharmacological Characters of *Pelargonium graveolens* Essential Oil from Palestine. Molecules.

[22] Boukhatem M.N., Kameli A., Ferhat M.A., Saidi F., Mekarnia M. (2013). Rose Geranium Essential Oil as a Source of New and Safe Anti-Inflammatory Drugs. Libyan J. Med..

[23] Mori-mestanza D., Valqui-rojas I., Caetano A.C., Culqui-arce C., Cruz-lacerna R., Cayo-colca I.S., Castro-alayo E.M., Balcázar-zumaeta C.R. (2025). Physicochemical Properties of Nanoencapsulated Essential Oils: Optimizing D-Limonene Preservation. Polymers.

[24] Yousef H.A., Fahmy H.M., Arafa F.N., Abd Allah M.Y., Tawfik Y.M., El Halwany K.K., El-Ashmanty B.A., Al-anany F.S., Mohamed M.A., Bassily M.E. (2023). Nanotechnology in Pest Management: Advantages, Applications, and Challenges. Int. J. Trop. Insect Sci..

[25] Wilson R.J., Li Y., Yang G., Zhao C.X. (2022). Nanoemulsions for Drug Delivery. Particuology.

[26] Sahu U., Malik T., Ibrahim S.S., Vendan S.E., Karthik P. (2022). Pest Management with Green Nanoemulsions. Bio-Based Nanoemulsions Agri-Food Appl..

[27] Kumar M., Bishnoi R.S., Shukla A.K., Jain C.P. (2019). Techniques for Formulation of Nanoemulsion Drug Delivery System: A Review. Prev. Nutr. Food Sci..

[28] Singh I.R., Pulikkal A.K. (2022). Preparation, Stability and Biological Activity of Essential Oil-Based Nano Emulsions: A Comprehensive Review. OpenNano.

[29] Chang Y., McLandsborough L., McClements D.J. (2013). Physicochemical Properties and Antimicrobial Efficacy of Carvacrol Nanoemulsions Formed by Spontaneous Emulsification. J. Agric. Food Chem..

[30] Yang Y., Marshall-Breton C., Leser M.E., Sher A.A., McClements D.J. (2012). Fabrication of Ultrafine Edible Emulsions: Comparison of High-Energy and Low-Energy Homogenization Methods. Food Hydrocoll..

[31] Vega-Vásquez P., Mosier N.S., Irudayaraj J. (2021). Hormesis-Inducing Essential Oil Nanodelivery System Protects Plants against Broad Host-Range Necrotrophs. ACS Nano.

[32] Yuliani S., Wahyuningsih K., Hernani, Herawati H., Hoerudin, Rahmini, Noveriza R. (2023). Spontaneous Emulsification of Citronella Oil: Effect of Processing Conditions and Production Scale. IOP Conf. Ser. Earth Environ. Sci..

[33] Giunti G., Campolo O., Laudani F., Zappalà L., Palmeri V. (2021). Bioactivity of Essential Oil-Based Nano-Biopesticides toward *Rhyzopertha dominica* (Coleoptera: Bostrichidae). Ind. Crops Prod..

[34] Hagag H., El-Sawah M.H.A., Raddy H.M., Gad H.A. (2024). Chemical Composition, Insecticidal Activities of *Origanum Majorana* L. Essential Oil Nanoemulsion against *Callosobruchus maculatus* and *Callosobruchus chinensis*. Egypt. J. Chem..

[35] Aisyah Y., Haryani S., Safriani N., El Husna N. (2018). Optimization of Emulsification Process Parameters of Cinnamon Oil Nanoemulsion. Int. J. Adv. Sci. Eng. Inf. Technol..

[36] Finney D.J. (1971). Probit Analysis. J. Pharm. Sci..

[37] Ostertag F., Weiss J., McClements D.J. (2012). Low-Energy Formation of Edible Nanoemulsions: Factors Influencing Droplet Size Produced by Emulsion Phase Inversion. J. Colloid Interface Sci..

[38] Tadros T., Izquierdo P., Esquena J., Solans C. (2004). Formation and Stability of Nano-Emulsions. Adv. Colloid Interface Sci..

[39] Saberi A.H., Fang Y., McClements D.J. (2013). Fabrication of Vitamin E-Enriched Nanoemulsions by Spontaneous Emulsification: Effect of Propylene Glycol and Ethanol on Formation, Stability, and Properties. Food Res. Int..

[40] Kelmann R.G., Kuminek G., Teixeira H.F., Koester L.S. (2007). Carbamazepine Parenteral Nanoemulsions Prepared by Spontaneous Emulsification Process. Int. J. Pharm..

[41] Marzuki N.H.C., Wahab R.A., Hamid M.A. (2019). An Overview of Nanoemulsion: Concepts of Development and Cosmeceutical Applications. Biotechnol. Biotechnol. Equip..

[42] Sharma U.C., Hariprasad P., Satya S. (2024). Efficacy Evaluation of *Eucalyptus Globulus* Essential Oil-Based Nanoemulsion- a Green Insecticide against *Callosobruchus maculatus*. Int. J. Trop. Insect Sci..

[43] Maurya A., Yadav A., Soni M., Paul K.K., Banjare U., Jha M.K., Dwivedy A.K., Dubey N.K. (2024). Nanoencapsulated Essential Oils for Post-Harvest Preservation of Stored Cereals: A Review. Foods.

[44] Draz K.A., Tabikha R.M., Eldosouky M.I., Darwish A.A., Abdelnasser M. (2022). Biotoxicity of Essential Oils and Their Nano-Emulsions against the Coleopteran Stored Product Insect Pests *Sitophilus oryzae* L. and *Tribolium castaneum* Herbst. Int. J. Pest Manag..

[45] Nenaah G.E., Ibrahim S.I.A., Al-Assiuty B.A. (2015). Chemical Composition, Insecticidal Activity and Persistence of Three Asteraceae Essential Oils and Their Nanoemulsions against *Callosobruchus maculatus* (F.). J. Stored Prod. Res..

[46] Turek C., Stintzing F.C. (2013). Stability of Essential Oils: A Review. Compr. Rev. Food Sci. Food Saf..

[47] de Oliveira J.L., Campos E.V.R., Bakshi M., Abhilash P.C., Fraceto L.F. (2014). Application of Nanotechnology for the Encapsulation of Botanical Insecticides for Sustainable Agriculture: Prospects and Promises. Biotechnol. Adv..

[48] Ibrahim S.S., Sahu U., Karthik P., Vendan S.E. (2023). Eugenol Nanoemulsion as Bio-Fumigant: Enhanced Insecticidal Activity against the Rice Weevil, *Sitophilus oryzae Adults*. J. Food Sci. Technol..

[49] Kedia A., Dubey N.K. (2018). Nanoencapsulation of Essential Oils: A Possible Way for an Eco-Friendly Strategy to Control Postharvest Spoilage of Food Commodities From Pests.

[50] Moura E.d.S., Faroni L.R.D.A., Zanuncio J.C., Heleno F.F., Prates L.H.F. (2019). Insecticidal Activity of *Vanillosmopsis arborea* Essential Oil and of Its Major Constituent α-Bisabolol against *Callosobruchus maculatus* (Coleoptera: Chrysomelidae). Sci. Rep..

[51] Hashem A.S., Awadalla S.S., Zayed G.M., Maggi F., Benelli G. (2018). *Pimpinella anisum* Essential Oil Nanoemulsions against *Tribolium castaneum*—Insecticidal Activity and Mode of Action. Environ. Sci. Pollut. Res..

[52] Ibrahim S.S. (2022). Polyethylene Glycol Nanocapsules Containing *Syzygium aromaticum* Essential Oil for the Management of Lesser Grain Borer, *Rhyzopertha dominica*. Food Biophys..

[53] Louni M., Shakarami J., Negahban M. (2018). Insecticidal Efficacy of Nanoemulsion Containing *Mentha longifolia* Essential Oil against *Ephestia kuehniella* (Lepidoptera: Pyralidae). J. Crop Prot..

[54] Ziaee M., Moharramipour S., Mohsenifar A. (2014). MA-Chitosan Nanogel Loaded with *Cuminum cyminum* Essential Oil for Efficient Management of Two Stored Product Beetle Pests. J. Pest Sci..

