# Supplementary material for: Geranium Oil Nanoemulsion Delivers More Potent and Persistent Fumigant Control of Callosobruchus maculatus in Stored Grain
Source: Foods. 2025 Oct 15;14(20):3514. doi: 10.3390/foods14203514 (PMC12564152; doi:10.3390/foods14203514)
Supplement: Supplementary file 1 [file foods-14-03514-s001.zip › foods-3896428-supplementary.pdf]

Supplementary material

Table S1. Average mortality (%) of *C. maculatus* adults exposed to bulk geranium essential oil (GOB) and geranium oil nanoemulsion (GONE) across various concentrations and exposure times.

Treatments	Average mortality (%) t^{a}(df), p-value				F^{b}(df), p- value
	24h	48h	72h	96h	
GOB-50 µL/L air<br>GONE-50 µL/L air	-0.86(18), p=0.40	-0.30(18), p=0.76	-1.54(18), p=0.14	-2.75(18), p=0.01	58.12(3), p<0.001<br>184.53(3), p<0.001
GOB-100 µL/L air<br>GONE-100 µL/L air	0.36(18), p=0.72	-1.13(18), p=0.27	-1.69(18), p=0.10	-5.33(18), p<0.001	34.63(3), p<0.001<br>69.78(3), p<0.001
GOB-150 µL/L air<br>GONE-150 µL/L air	-0.28(18), p=0.77	-1.58(18), p=0.12	-2.48(18), p=0.02	-4.25(18), p<0.001	19.32(3), p<0.001<br>177.81(3), p<0.001
GOB-200 µL/L air<br>GONE-200 µL/L air	0.34(18), p=0.73	-2.16(18), p=0.04	-4.58(18), p<0.001	-1.89(18), p=0.07	36.23(3), p<0.001<br>389.41(3), p<0.001
GOB-250 µL/L air<br>GONE-250 µL/L air	-0.52(18), p=0.60	-1.34(18), p=0.19	-1.96(18), p=0.06	-0.99(18), p=0.33	19.40(3), p<0.001<br>383.03(3), p<0.001

[a] Values are based on independent samples *t*-tests comparing GOB and GONE at corresponding concentrations and time points.

[b] Post-hoc comparisons using Tukey's HSD test for mean separation at a significant level of $p < 0.05$ were conducted.

Table S2. Average mortality (%) of *C. maculatus* adults exposed to bulk geranium essential oil (GOB) and geranium oil nanoemulsion (GONE) across various concentrations and exposure times.

	Average mortality (%)			
**Treatments**	**24h**	**48h**	**72h**	**96h**
Additive-only control	-	-	-	9.00±1.00*
Control- 0 µL/L air	0.00±0.00e†	3.00±1.52c	6.00±2.21d	10.00±1.49e
GOB-50 µL/L air	18.00±2.00d	41.00±1.79b	73.00±4.22c	78.00±3.59d
GOB-100 µL/L air	46.00±2.21c	65.00±2.23a	80.00±2.98bc	84.00±2.21cd
GOB-150 µL/L air	56.00±2.21b	70.00±2.58a	86.00±4.52abc	91.00±2.33bc
GOB-200 µL/L air	64.00±2.21ab	74.00±3.05a	93.00±1.52ab	96.00±2.21ab
GOB-250 µL/L air	68.00±2.91a	76.00±7.91a	97.00±1.52a	99.00±1.00a
**F (df), *p*-value**	220.41(5), *p*<0.001	53.95(5), *p*<0.001	67.66(5), *p*<0.001	122.11(5), *p*<0.001
Control- 0 µL/L air	0.00±0.00e	3.00±1.52e	6.00±2.21c	10.00±1.49c
GONE-50 µL/L air	20.00±1.49d	42.00±2.49d	83.00±2.13b	89.00±1.79b
GONE-100 µL/L air	45.00±1.67c	69.00±2.76c	88.00±2.90b	97.00±1.52a
GONE-150 µL/L air	57.00±2.60b	75.00±1.66bc	98.00±1.33a	100.00±0.00a
GONE-200 µL/L air	63.00±2.13ab	82.00±1.33ab	100.00±0.00a	100.00±0.00a
GONE-250 µL/L air	70.00±1.49a	88.00±1.33a	100.00±0.00a	100.00±0.00a
**F (df), *p*-value**	344.25(5), *p*<0.001	231.25(5), *p*<0.001	213.58(5), *p*<0.001	374.34(5), *p*<0.001

* Additive-only control (formulation without geranium oil, 250 µL/L air, 96 h exposure) showed no significant difference from the negative control ($p = 0.58$). †All data are means ± standard error of means (SEM). Under the same time and among bulk geranium oil (GOB) or nanoemulsion (GONE), means followed by the same letters are not significantly different ($p<0.05$). Post-hoc comparisons using Tukey's HSD test for mean separation at a significance level of $p < 0.05$ were conducted.

Table S3. Reproductive Effects of Geranium Oil (GOB) and Its Nanoemulsion (GONE) on *C. maculatus*: Average Egg Production by a single female and Adult Emergence 45 days post-treatment across concentrations and exposure times.

	Eggs laid by a single female t^a(df), *p*-value				Adult emergence (percentage) t^a(df), *p*-value			
**Treatments**	24h	48h	72h	96h	24h	48h	72h	96h
GOB-50 µL/L air<br>GONE-50 µL/L air	1.67(18), *p*=0.11	1.50(18), *p*=0.14	4.72(18), *p*<0.001	1.96(18), *p*=0.06	1.19(18), *p*=0.24	1.20(18), *p*=0.24	3.43(18), *p*=0.002	5.44(18), *p*<0.001
GOB-100 µL/L air<br>GONE-100 µL/L air	-0.56(18), *p*=0.58	0.73(18), *p*=0.47	1.65(18), *p*=0.11	1.67(18), *p*=0.11	0.11(18), *p*=0.90	0.17(18), *p*=0.86	8.64(18), *p*<0.001	11.19(18), *p*<0.001
GOB-150 µL/L air<br>GONE-150 µL/L air	1.29(18), *p*=0.21	1.99(18), *p*=0.06	2.53(18), *p*=0.02	0.01(18), *p*=0.98	2.68(18), *p*=0.01	2.76(18), *p*=0.01	6.45(18), *p*<0.001	17.61(18), *p*<0.001
GOB-200 µL/L air<br>GONE-200 µL/L air	-0.63(18), *p*=0.53	-0.50(18), *p*=0.61	0.92(18), *p*=0.36	-0.35(18), *p*=0.72	2.36(18), *p*=0.02	1.95(18), *p*=0.06	16.79(18), *p*<0.001	13.92(18), *p*<0.001
GOB-250 µL/L air<br>GONE-250 µL/L air	1.78(18), *p*=0.09	0.35(18), *p*=0.73	0.19(18), *p*=0.84	0.40(18), *p*=0.69	2.71(18), *p*=0.01	3.03(18), *p*=0.007	15.35(18), *p*<0.001	-

[a] Values are based on independent samples *t*-tests comparing GOB and GONE at corresponding concentrations and time points.

Table S4. Reproductive Effects of Geranium Oil (GOB) and Its Nanoemulsion (GONE) on *C. maculatus*: Average Egg Production by a single female and Adult Emergence 45 days post-treatment across concentrations and exposure times.

	**Eggs laid by a single female**				**Adult emergence (percentage)**			
**Treatments**	24h	48h	72h	96h	24h	48h	72h	96h
Control-0 µL/L air	11.20±0.42a	15.10±0.31a	21.40±0.65a	23.70±0.52a	85.03±1.30a	84.31±1.15a	82.64±1.59a	78.15±1.28a
GOB-50 µL/L air	10.80±0.39a	10.40±0.27b	10.90±0.41b	10.70±0.26b	74.26±2.22b	70.23±1.04b	60.70±1.88b	44.05±1.68b
GOB-100 µL/L air	8.40±0.31b	8.10±0.31c	7.80±0.42c	7.00±0.42c	72.49±1.21b	68.67±2.4bc	59.57±2.04b	25.15±2.02c
GOB-150 µL/L air	7.90±0.31bc	8.00±0.33c	7.20±0.36cd	5.90±0.35c	70.06±2.29b	67.60±1.97bc	52.89±3.41b	17.87±1.53d
GOB-200 µL/L air	7.10±0.23c	7.20±0.29c	6.60±0.43cd	6.20±0.33c	68.73±2.47b	66.24±2.80bc	41.14±2.19c	15.94±1.68d
GOB-250 µL/L air	6.80±0.25c	7.10±0.41c	6.10±0.35d	6.70±0.33c	66.77±1.56b	60.28±2.01c	27.28±2.08d	0.00±0.00e
**F (df), *p*-value**	34.12(5), *p*<0.001	75.78(5), *p*<0.001	137.37(5), *p*<0.001	217.99(5), *p*<0.001	12.70(5), *p*<0.001	17.60(5), *p*<0.001	69.89(5), *p*<0.001	325.91(5), *p*<0.001
Control-0 µL/L air	11.20±0.42a	15.10±0.31a	21.40±0.65a	23.70±0.52a	85.03±1.30a	84.31±1.15a	82.64±1.59a	78.15±1.28a
GONE-50 µL/L air	9.90±0.38ab	9.70±0.40b	8.50±0.31b	9.90±0.31b	71.46±1.00b	68.45±1.03b	49.87±2.52b	29.20±2.08b
GONE-100 µL/L air	8.60±0.22bc	7.80±0.25c	6.90±0.35c	6.10±0.31c	72.19±1.72b	68.08±2.50b	38.59±1.29c	2.42±0.71c
GONE-150 µL/L air	7.40±0.22cd	7.20±0.20c	6.00±0.33c	5.90±0.41c	62.62±1.52c	60.56±1.59bc	28.37±1.63d	0.00±0.00d
GONE-200 µL/L air	7.30±0.21d	7.20±0.43c	6.10±0.35c	6.40±0.40c	61.96±1.44c	58.48±2.76c	5.66±0.74e	0.00±0.00d
GONE-250 µL/L air	6.10±0.31e	6.90±0.38c	6.00±0.30c	6.50±0.48c	59.60±2.15c	52.57±1.57c	0.95±0.49f	0.00±0.00d
**F (df), *p*-value**	38.86(5), *p*<0.001	83.87(5), *p*<0.001	179.05(5), *p*<0.001	182.85(5), *p*<0.001	40.87(5), *p*<0.001	37.75(5), *p*<0.001	404.13(5), *p*<0.001	808.32(5), *p*<0.001

[*]All data are means ± standard error of means (SEM). Under the same time and among bulk geranium oil (GOB) or nanoemulsion (GONE), means followed by the same letters in the same column are not significantly different (*p*<0.05)

Post-hoc comparisons using Tukey's HSD test for mean separation at a significance level of $p < 0.05$ were conducted.

Table S5. Persistent fumigant toxicity of geranium essential oil (GOB) and geranium oil nanoemulsion (GONE) at LC90 on C. maculatus adults.

	t*-test for Equality of Means		
Exposure point	t	df	*p*-value
D2	-.767	18	0.453
D4	-2.135	18	0.047
D6	-4.291	18	0.001
D8	-4.019	18	0.001
D10	-8.231	18	0.001
D12	-6.820	18	0.001
D14	-7.016	18	0.001
D16	-8.618	18	0.001
D18	-12.933	18	0.001
D20	-13.613	18	0.001
D22	-4.583	18	0.001
D24	-1.456	18	0.162

*Significant differences between treatments were tested by comparing means using a t-test, $p<0.05$.

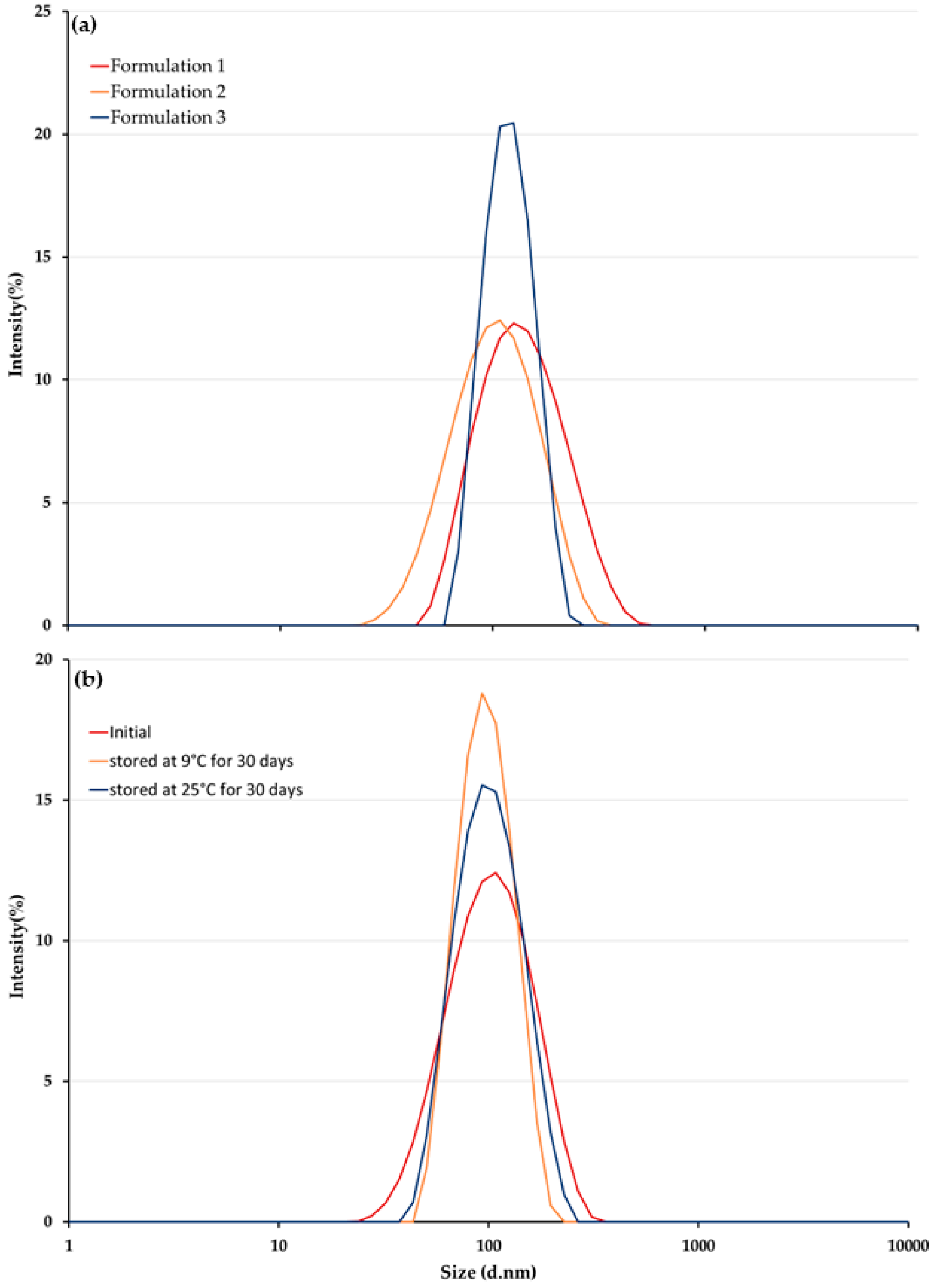

Figure S1. (a) Size distribution by intensity of geranium oil nanoemulsion (GONE) across three formulations; (b) Size distribution by intensity of formulation 2 (F2) during 30 days of storage at different temperatures.

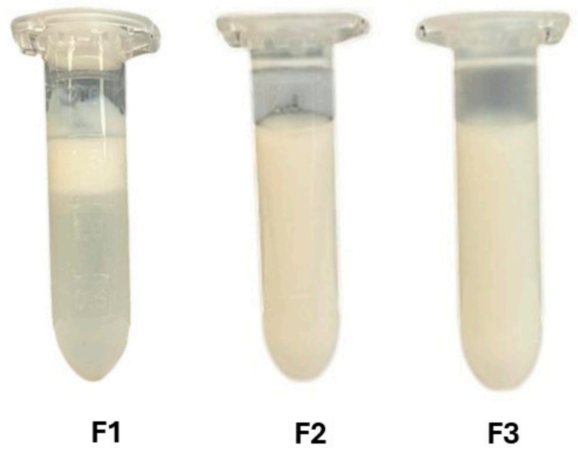

Figure S2. Visual appearance of geranium oil nanoemulsion (GONE) formulations (F1, F2, and F3). F1, F2, and F3 correspond to formulations containing 5%, 10%, and 15% Tween-80, respectively. Phase separation was observed in F1.
